# The Influence of Vitamin D Intake and Status on Mental Health in Children: A Systematic Review

**DOI:** 10.3390/nu13030952

**Published:** 2021-03-16

**Authors:** Dominika Głąbska, Aleksandra Kołota, Katarzyna Lachowicz, Dominika Skolmowska, Małgorzata Stachoń, Dominika Guzek

**Affiliations:** 1 Department of Dietetics, Institute of Human Nutrition Sciences, Warsaw University of Life Sciences (WULS-SGGW), 159C Nowoursynowska Street, 02-776 Warsaw, Poland; aleksandra_kolota@sggw.edu.pl (A.K.); katarzyna_lachowicz@sggw.edu.pl (K.L.); dominika_skolmowska@sggw.edu.pl (D.S.); malgorzata_stachon@sggw.edu.pl (M.S.); 2 Department of Food Market and Consumer Research, Institute of Human Nutrition Sciences, Warsaw University of Life Sciences (WULS-SGGW), 159C Nowoursynowska Street, 02-776 Warsaw, Poland; dominika_guzek@sggw.edu.pl

**Keywords:** vitamin D, intake, status, supplementation, mental disorders, mental health, depression

## Abstract

A potential role of vitamin D in some components of mental health is currently suggested, but the analyses are conducted mainly for adults, while for young individuals mental health is especially important, due to its lifelong effects. The aim of the study was to analyze the association between vitamin D intake or status and mental health in children within a systematic review of literature, including both intervention and observational studies. The literature search was conducted according to the PRISMA guidelines and it covered peer-reviewed studies included in databases of PubMed and Web of Science until October 2019. The studies presenting either vitamin D intake, or vitamin D status in human subjects were allowed (excluding subjects with intellectual disabilities, eating disorders and neurological disorders), while for mental health the various methods of assessment and wide scope of factors were included. The bias was assessed using the Newcastle–Ottawa Scale (NOS). The review was registered in the PROSPERO database (CRD42020155779). A number of 7613 studies after duplicate removing were extracted by two independent researchers, followed by screening and assessment for eligibility, conducted by two independent researchers in two steps (based on title and abstract). Afterwards, the full texts were obtained and after reviewing, a number of 24 studies were included. The synthetic description of the results was prepared, structured around exposure (vitamin D supplementation/status) and outcome (components of mental health). The included studies were conducted either in groups of healthy individuals, or individuals with mental health problems, and they assessed following issues: behavior problems, violence behaviors, anxiety, depressive symptoms/depression, aggressive disorder, psychotic features, bipolar disorder, obsessive compulsive disorder, suicidal incident, as well as general patterns, as follows: mental health, level of distress, quality of life, well-being, mood, sleep patterns. The vast majority of assessed studies, including the most prominent ones (based on the NOS score) supported potential positive influence of vitamin D on mental health in children. As a limitation of the analysis, it should be indicated that studies conducted so far presented various studied groups, outcomes and psychological measures, so more studies are necessary to facilitate comparisons and deepen the observations. Nevertheless, vitamin D intake within a properly balanced diet or as a supplementation, except for a safe sun exposure, should be indicated as an element supporting mental health in children, so it should be recommended to meet the required 25(OH)cholecalciferol blood level in order to prevent or alleviate mental health problems.

## 1. Introduction

The number of studies analyzing vitamin D status, relevance of its supplementation, as well as a link between this nutrient and clinical outcomes is currently increasing [1]. The knowledge about vitamin D is still broadening [2], but various serum 25-hydroxyvitamin D (25(OH)D) level thresholds are defined by prominent authorities as vitamin D deficiency, namely lower than 30 nmol/L [3,4] and lower than 50 nmol/L [5,6]. The prevalence of vitamin D deficiency, depending on 25(OH)D level threshold is estimated as 13.0% and 40.4%, respectively for 30 and 50 nmol/L, irrespective of age group, ethnic mix, and latitude of study population [7]. In a vast majority of European countries, there is a common problem with achieving the recommended vitamin D status [8], except for Finland, due to high fish intake in this country, combined with food fortification and supplementation [9], which is applied within national program since 2003 in this country [10].

Such insufficient intake of vitamin D may result in a number of health-related consequences, including not only osteoporosis [11], but also other diseases and conditions. Recent meta-analyses indicated that vitamin D may reduce cancer mortality [12], as well as all-cause mortality [13]. Similarly, other meta-analyses emphasized its role for cardiac outcomes in coronary artery disease patients [14], prevention of diabetes [15] and prevention of acute respiratory infections [16], as well as experienced pain [17] and migraines [18]. Even for the COVID-19 it was stated that vitamin D may be associated with the risk [19] and severity of infection [20].

Among other diseases which are studied, as potentially influenced by vitamin D status, are those associated with broad area of mental health. The meta-analysis of randomized controlled trials by Cheng et al. [21] indicated that vitamin D supplementation can reduce negative emotions. Similarly, the systematic review by Hoffmann et al. [22] indicated that vitamin D supplementation may have a small to moderate effect on health-related quality of life, which was hypothesized by authors as resulting from widespread roles of this nutrient throughout the body and its association with many chronic diseases and mental health. At the same time, the meta-analyses by Gowda et al. [23] and by Li et al. [24] indicated that vitamin D supplementation did not cause significant reduction in depression. However, the meta-analysis by Spedding [25] indicated that such observations may result from biological flaws of primary studies, as in case of studies which he identified as those without biological flaws, he observed that vitamin D supplementation caused statistically significant improvement in depression. Similarly, the meta-analysis of randomized controlled trials by Vellekkatt and Menon [26] indicated that vitamin D supplementation favorably impacted depression ratings in major depression. Also the meta-analyses by Anglin et al. [27] and by Ju et al. [28] demonstrated that low vitamin D blood concentration may be associated with depression. At the same time, for the depressive symptoms, the meta-analysis by Shaffer et al. [29] indicated that vitamin D supplementation may be effective in reducing depressive symptoms in patients with clinically significant depression.

As indicated, there were some analysis conducted for effects of vitamin D on depression or depressive symptoms [23,24,25,26,27,28,29], as well as single studies for negative emotions [21] and for quality of life [22], but not for other aspects of the broad spectrum of mental health. According to the definition by the World Health Organization (WHO), mental health is interpreted as a state of well-being in which the individuals realize their own abilities, can cope with the normal stresses of life, can work productively and fruitfully, and are able to make a contribution to their community [30]. Based on the indicated scope of mental health, not only depression, negative emotions, or quality of life are commonly included while analyzing mental health, but also symptoms such as stress, nervousness, anxiety, self-efficacy, self-esteem, happiness, or general well-being, life satisfaction, and mood [31]. Moreover, it should be indicated that while there are some studies for adults, there are no such analysis conducted for children or adolescents, in spite of the fact that their mental health is especially important, as it has lifelong effects on individuals and society [32]. Some studies conducted so far revealed that children with psychiatric disorders might have higher prevalence of hypovitaminosis D than the general pediatric population [33] and that the association observed in adults may be also noticed in children and adolescents [34].

Based on the presented background, the aim of the study was to analyze the association between vitamin D intake or status and mental health in children within a systematic review of literature, including both intervention and observational studies.

## 2. Materials and Methods

### 2.1. Design

The literature search was conducted according to the guidelines of the Preferred Reporting Items for Systematic Reviews and Meta-Analyses (PRISMA) [35] and it covered peer-reviewed studies included in databases of PubMed and Web of Science until October 2019. The review was registered in the International Prospective Register of Systematic Reviews (PROSPERO) database (CRD42020155779).

### 2.2. Inclusion and Exclusion Criteria

The included observational studies presented association between vitamin D intake (either from diet or supplementation) and mental health in children. The inclusion criteria were as follows:

(1) studies conducted in children/adolescents;
(2) studies presenting vitamin D intake (either from diet or supplementation), or vitamin D status assessed (e.g., 25(OH)cholecalciferol blood level);
(3) studies presenting mental health, while various methods of assessment (e.g., medical diagnosis, questionnaire) and wide scope of factors associated with mental health were allowed.

The exclusion criteria were as follows:

(1) animal model studies;
(2) studies presenting influence of maternal vitamin D intake/status on mental health of their offspring;
(3) studies presenting influence of broad spectrum of nutrients combined;
(4) studies in participants with intellectual disabilities;
(5) studies in participants with eating disorders;
(6) studies in participants with neurological disorders (e.g., epilepsy).

The studies to be included were to be published in English in a peer-reviewed journal and were allowed to be conducted in any country, with no other criteria based on location or characteristics of the studied sample.

### 2.3. Searching Strategy

The literature searching covered intervention and observational studies included in databases of PubMed and Web of Science until October 2019. The search was based on the potential outcomes, commonly included to the systematic reviews [31]. The applied detailed electronic search strategy is presented in Table 1. As various outcomes were studied, the results of the systematic review were impossible to be reanalyzed as a meta-analysis, so synthetic description of results was prepared and structured around exposure (vitamin D intake/status) and outcome (components of mental health).

**Figure 1:**
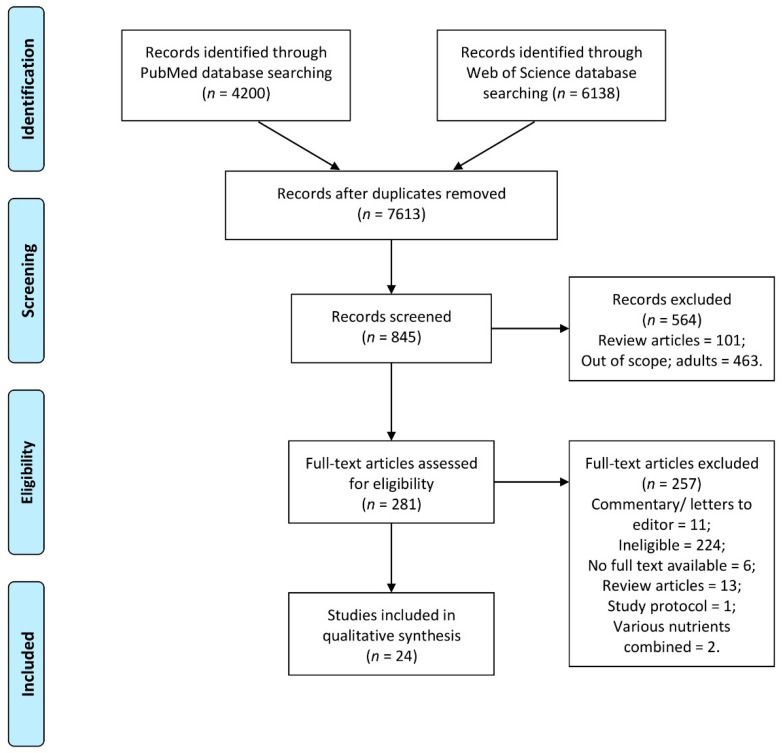
The detailed inclusion procedure in a systematic review of the literature.

Identified studies after duplicate removing were extracted by two independent researchers followed by screening and assessment for eligibility, conducted by two independent researchers in two steps (to verify it separately based on title and in case of included, also based on abstract). If any disagreement appeared it was discussed with other researchers. Meanwhile, potentially eligible studies which were unavailable, were obtained by contacting the corresponding author of the study to ask them for a full text. Finally, the full texts were analyzed by two independent researchers. If any disagreement appeared it was discussed with other researchers. The detailed inclusion procedure is presented in Figure 1.

In case of one included study, due to the fact that it was presented as an abstract only [36], the manual search for the full text article was conducted to replace abstract by full text [37].

### 2.4. Data Extraction Procedure

The data extracting was conducted by two independent researchers. If any disagreement appeared it was discussed with other researchers. If any data were missing, they were requested by contacting the corresponding authors of the study to ask them for a detailed information. In case of data provided on request, they are referred in Results section as provided on request. The data were extracted based on the common approach to extract the following information:

(1) general characteristics of the study, including: authors, design of the study, country or region; studied group; studied period;
(2) participants of the study, including: number of participants, sex, age, inclusion and exclusion criteria;
(3) assessment of vitamin D intake, or status, including: applied supplementation, or method of assessment of vitamin D status;
(4) assessment of mental health, including: method of assessment, applied psychological measure;
(5) findings of the study, including: observations described by authors of the study; findings formulated by the authors of the study.

The risk of bias and methodological quality of the included studies was assessed as recommended based on the Cochrane guidelines [38] using the Newcastle-Ottawa Scale (NOS) [39]. The case control studies were assessed, including the following criteria: selection, comparability, and exposure, while cohort studies were assessed, including the following criteria: selection, comparability, and outcome. The total score was described while compared with the following categories: very high (from 0 to 3 points), high (from 4 to 6 points), and low risk of bias (from 7 to 9 points), as it is commonly applied [40].

## 3. Results

### 3.1. Intervention Studies

The basic characteristics of the included intervention studies [37,41,42,43,44,45,46], including design of study, location, studied group and time are described in Table 2. The included intervention studies were conducted in various countries—United States of America [37,45], Nordic countries [43,46], Iran [41,42] and Turkey [44]. Only one study described a single sex sample of adolescent girls [42], while other studies were conducted in a mixed populations of boys and girls of various age [37,41,43,44,45,46]. Moreover, a majority of intervention studies assessed a specific populations of children with a sickle cell disease [37], attention deficit hyperactivity disorder (ADHD) [41], autism spectrum disorders [44], bipolar spectrum disorders [45] and depression [46], being in some studies compared with control groups.

The characteristics of the subjects of the included intervention studies are described in Table 3. The included intervention studies were conducted in samples of various size, differing from 35 [45] to 940 participants [42], while the inclusion and exclusion criteria were formulated to obtain required samples. For the vast majority of studies, the exclusion criteria included any condition that would interfere with planned vitamin D supplementation, including the previously applied supplementation [37,41,42,43,44], participation in any other study impacting serum 25(OH)cholecalciferol blood level [37], and any disorder interfering with the action, absorption, distribution, metabolism or excretion of vitamin D [45], or required being stably medicated if taking daily supplementation [45].

The exposure, intervention and outcome of the included intervention studies are described in Table 4. The included studies presented various models of intervention, mainly conducted for 3 months [37,41,43,44,46], but also 2 months [45] and 9 weeks [42], while applied vitamin D doses differed from 25 µg per day [41] to 1250 µg per week (179 µg per day) [42,44]. The observed outcomes were associated with depressive symptoms/depression [42,45], aggressive disorder [42], suicidal incident [45], as well as general patterns, as follows: mental health [41,43], quality of life [37], well-being [46], mood [45,46], sleep patterns [44], which were assessed while using dedicated psychological measures.

The findings presented in the intervention studies are described in Table 5, based on the data presented by authors of the refereed studies as the most important ones.

### 3.2. Observational Studies

The basic characteristics of the included observational studies [47,48,49,50,51,52,53,54,55,56,57,58,59,60,61,62,63], including study design, location, studied group and time are described in Table 6. The included observational studies were conducted in various countries—United States of America [51,59,60,61], United Kingdom [62,63], Germany [54,57], Turkey [52,53,56], China [48,49,50], as well as Colombia [47], Canada [55] and Iran [58]. All the studies were conducted in a mixed populations of boys and girls of various age [47,48,49,50,51,52,53,54,55,56,57,58,59,60,61,62,63]. Some observational studies assessed a specific populations of children with a stable asthma [48], on chronic hemodialysis or peritoneal dialysis [49], with depressive disorders [50], mood symptom changes and elevated symptoms of mania [51], obsessive-compulsive disorder [52,53], established mental health diagnosis [55], cystic fibrosis [59], major depressive disorder [60], and on acute mental health treatment [61], being in some studies compared with control groups. 

The characteristics of the participants of the included observational studies are described in Table 7. The included observational studies were conducted in the samples of various size, differing from 36 [51] to 9068 participants [54], while the inclusion and exclusion criteria were formulated to obtain homogenic samples to obtain the aim of the study. In case of some studies, the exclusion criteria included the previously applied vitamin D supplementation [48,49,52].

The exposure, intervention and outcome of the included observational studies are described in Table 8. The included studies presented the influence of 25(OH)cholecalciferol blood level on various mental health related outcomes. The observed outcomes were associated with behavior problems [47], violence behaviors [58], anxiety [49,53], depressive symptoms/depression [50,51,52,53,56,59,60,63], psychotic features [61], bipolar disorder [51], obsessive compulsive disorder [52,53], as well as general patterns, as follows: mental health [54,55,62], level of distress [57,58], quality of life [48,57], which were assessed while using dedicated psychological measures.

The findings presented in the observational studies are described in Table 9, based on the data presented by authors of the refereed studies as the most important ones.

### 3.3. Summary

The summary of observations and conclusions for included studies of association between vitamin D and mental health, with the total NOS score are described in Table 10. It was observed that for the vast majority of included studies, both intervention and observational ones, the results supported beneficial association. Only in case of 2 studies, no effect of vitamin D was stated for bipolar disorder and major depressive disorder [51] and mental health (assessed using DSM-V criteria) [55]. In case of three studies the effect of vitamin D was inconclusive, as it was observed only for some of applied analysis [50,60] or depending on the studied component of obsessive compulsive disorder [52]. However, while the total NOS score is taken into account, it should be indicated that all the studies of low risk of bias support the positive effect of vitamin D [47,62,63].

## 4. Discussion

In spite of the fact that studies of association between vitamin D and mental health present various studied groups, outcomes and psychological measures, the observed results are consistent and they suggest potential beneficial effect of vitamin D blood level or applied supplementation on mental health. Taking this into account, it may be indicated that regardless of the studied group and studied effect associated with mental health, the vitamin D is crucial for mental health.

The association between vitamin D and mental health was so far studied mainly for depression or depressive symptoms [23,24,25,26,27,28,29] and some potential mechanisms explaining the influence of vitamin D were supposed [64]. Vitamin D has potential to cross the blood-brain barrier, to activate receptors in brain cells and to exert its direct impact in the central nervous system [65]. Moreover, there is some evidence for the link between vitamin D and Vitamin D Receptors (VDRs) and the regulation of human behavior, that is strongly suggested by the presence of VDRs in such brain areas as cortex, cerebellum and limbic system [66]. At the same time, Eyles et al. [67] reported that the mechanism potentially important in neuroendocrine functioning may be associated with the VDRs in the hypothalamus. However, it should be also mentioned that VDR genes are polymorphic and their variations occur frequently, what can cause various vitamin D-related dysfunctions [68].

Moreover, studies in animal models indicated potential anti-inflammatory effects of vitamin D administration in hippocampus and hypothalamus and its modulating effects on brain-derived neurotropic factor (BDNF) [65] which may also play a role. Simultaneously, the role of vitamin D may be attributed to its neuroprotective role in the brain which is reflected in modulating neurotrophic signaling [69], and in regulating inflammation by inhibiting proinflammatory cytokines [70].

Taking into account the potential mechanisms described above and the results of the recent studies suggesting beneficial effect of vitamin D on mental health, this area is indicated within the current research perspectives associated with vitamin D [71]. Regardless of the fact, that biological mechanisms linking vitamin D and mental health are still not fully understood [72], it should be indicated that vitamin D may have beneficial effects, which is important considering high prevalence of mental health problems which is not decreasing, despite a substantial increases in the provision of treatment [73]. Taking this into account, improving vitamin D status by applying adequate intake either within a properly balanced diet or as a supplementation, may be beneficial also for the prevention and treatment of mental health problems in children.

The included studies analyzing the association between vitamin D intake or status and mental health in children assessed various aspects of mental health, so based on the presented observations, it may be suggested that vitamin D is associated with the broad area of mental health with all its elements. However, the limitations of the presented systematic review must be also described. The most important fact results from the limited number of studies published so far, while included studies presented various studied groups, outcomes and psychological measures, so more studies are necessary to deepen the observations. Moreover, since the studies assessed a wide range of possible effects, meta-analysis was impossible [74], so only the systematic review was conducted. Last but not least, only peer-reviewed studies included in databases of PubMed and Web of Science are presented in the systematic review which may have caused that some interesting results are not presented.

At the same time, it should be indicated that some researchers suggest the possible reverse causality in the association between vitamin D and mental health [75]. It results from the fact that some individuals with mental health problems, including depression, may avoid outdoor activity and have poor appetite, resulting in reduced sunlight exposure and consequently reduced endogenous vitamin D synthesis, as well as reduced dietary vitamin D intake [76]. At the same time, they may have increased demand for vitamin D resulting from the disturbed calcium homeostasis [64] observed in patients with mental health problems [77].

Based on the prepared systematic review, vitamin D intake within a properly balanced diet or as a supplementation, except for a safe sun exposure, should be indicated as an element supporting mental health in children, so it should be recommended to meet the required 25(OH)cholecalciferol blood level in order to prevent or alleviate mental health problems.

## 5. Conclusions

The vast majority of assessed studies, including the most prominent ones (based on the NOS score) supported potential positive influence of vitamin D on mental health in children. Vitamin D intake within a properly balanced diet or as a supplementation, except for a safe sun exposure, should be indicated as an element supporting mental health in children, so it should be recommended to meet the required 25(OH)cholecalciferol blood level in order to prevent or alleviate mental health problems.

## Figures and Tables

**Table 1:** The applied detailed electronic search strategy for databases of PubMed and Web of Science.

Database	The Applied Full Electronic Search Strategy
PubMed	(((mental health[T/A] OR mental disorders[T/A] OR mental disorder[T/A] OR psychological distress[T/A] OR mood disorders[T/A] OR depression[T/A] OR suicidal[T/A] OR suicide[T/A] OR anxiety[T/A] OR well-being[T/A] OR wellbeing[T/A] OR quality of life[T/A] OR self esteem[T/A] OR self-esteem[T/A] OR self efficacy[T/A] OR self-efficacy[T/A] OR resilience[T/A] OR empowerment[T/A] OR social participation[T/A] OR mental capital[T/A] OR life skills[T/A] OR emotional[T/A] OR psychology[T/A] OR psychosocial[T/A] OR psychiatry[T/A])) AND (vitamin D[T/A] OR vitamin D2[T/A] OR vitaminD3[T/A] OR D2[T/A] OR D3[T/A] OR ergocalciferol[T/A] OR cholecalciferol[T/A] OR 25-hydroxyvitamin D[T/A] OR 3-epi-25hydroxyvitaminD[T/A] OR calcitriol[T/A] OR dihydroxycholecalciferol[T/A])) NOT (animal NOT (animal AND human)[MeSH Terms])
Web of Science	(TS=(“vitamin D” OR “vitamin D2” OR “vitamin D3” OR “D2” OR “D3” OR “ergocalciferol” OR “cholecalciferol” OR “25-hydroxyvitamin D” OR “3-epi-25 hydroxyvitamin D” OR “calcitriol” OR “dihydroxycholecalciferol”) AND TS=(“mental health” OR “mental disorders” OR “mental disorder” OR “psychological distress” OR “mood disorder” OR “depression” OR “suicidal” OR “suicide” OR “anxiety” OR “well-being” OR “wellbeing” OR “quality of life” OR “self esteem” OR “self-esteem” OR “self efficacy” OR “self-efficacy” OR “resilience” OR “empowerment” OR “social participation” OR “mental capital” OR “life skills” OR “emotional” OR “psychology” OR “psychosocial” OR “psychiatry”) NOT TS=(“animal” NOT (“animal” AND “human”)))

T/A—Title/Abstract.

**Table 2:** The basic characteristics of the included intervention studies, including study design, location, studied group and time.

Ref.	Authors, Year	Design of Study	Location	Studied Group	Time
[37]	Dougherty et al., 2020	Randomized study	United States of America (USA)/Philadelphia	African American children aged 5–20 years with and without sickle cell disease	April 2012–January 2013
[41]	Naeini et al., 2019	Double-blind, randomized, controlled clinical trial	Iran/Isfahan	Students aged 6–13 years with ADHD	Not specified
[42]	Bahrami et al., 2018	Longitudinal study with intervention	Iran/Mashhad and Sabzevar	Adolescent girls	Not specified
[43]	Grung et al., 2017	Randomized double-blind placebo control trial	Norway/Bergen	Healthy volunteers aged 13–14 years	January–April 2014
[44]	Guler et al. 2016	Intervention study	Turkey/Istanbul	Children aged 4–10 years with autism spectrum disorders	April 2014–November 2015
[45]	Sikoglu et al., 2015	Intervention study within Child and Adolescents NeuroDevelopment Initiative (CANDI) at the University of Massachusetts Medical School	USA/Massachusetts	Children and adolescents with bipolar spectrum disorders from CANDI	Not specified
[46]	Högberg et al., 2012	Intervention study	Sweden	Depressed adolescents	Not specified

ADHD—Attention Deficit Hyperactivity Disorder.

**Table 3:** The characteristics of the subjects of the included intervention studies.

Ref.	Number of Participants (Girls)	Age (Years) (Mean with SD)	Inclusion Criteria/Exclusion Criteria
[37]	44 (22)	11.0 ± 4.0 for sickle cell disease group 10.0 ± 4.0 for healthy group	Inclusion: African American; aged 5−20 years; with and without sickle cell disease; recruited from the Comprehensive Sickle Cell Center at the Children’s Hospital of Philadelphia (CHOP); healthy subjects from the CHOP network of primary care centers and the greater Philadelphia region Exclusion: participation in another study impacting serum 25(OH)cholecalciferol (25(OH)D); pregnant or lactating females; other chronic conditions or use of medications affecting growth, dietary intake, or nutritional status; use of vitamin D to treat vitamin D deficiency; baseline elevated serum calcium concentration; taking supplements containing vitamin D; BMI > 85th percentile for age and sex; chronic transfusion therapy (for sickle cell disease group)
[41]	71 (12)	9.2 ± 1.8 for vitamin D supplementation group 9.0 ± 1.2 for placebo group	Inclusion: aged 6–13 years; diagnosed with ADHD Exclusion: poor compliance; BMI > 25; any apparent chronic disease; intake of vitamin D supplement, omega 3 or zinc during the past two months; use of non-pharmacological treatments such as neurofeedback; play therapy; any vision or movement disabilities; inability to provide informed consent
[42]	940 (940)	14.6 ± 1.5	Inclusion: absence of any autoimmune, cardiovascular, metabolic bone, thyroid, parathyroid, or adrenal disorder/disease; absence of hepatic failure, kidney diseases, malabsorption, or cancer Exclusion: any anti-inflammatory, antidepressant, antidiabetic, or anti-obesity drugs; vitamin D or calcium supplementation; hormone therapy during the past 6 months
[43]	50 (32)	14.0 *	Inclusion: healthy; aged 13–14 years, recruited from two schools in the demographic area in Bergen Exclusion: using nutrient supplements (e.g., cod liver oil, multivitamins)
[44]	120 (32)	7.1 ± 1.5 for autism spectrum disorder group 6.9 ± 1.6 for control group	Inclusion: meeting the diagnostic criteria for autistic disorder according to the DSM-V (for autism spectrum disorder group); mentally and neurologically healthy (for control group); age and sex-matched control group Exclusion: history of metabolic disorder, systemic inflammatory disease, obesity; usage of antiepileptic drugs, steroids, estrogens, immune suppressors, bisphosphonates, calcium, and vitamin D
[45]	35 (15)	12.3 ± 3.2 for bipolar spectrum disorder group 11.9 ± 3.6 for control group	Inclusion: children and adolescents from Child and Adolescents NeuroDevelopment Initiative (CANDI) at the University of Massachusetts Medical School; aged 6–17 years; being able ingest the vitamin D3 orally; the screen visit Young Mania Rating Scale (YMRS) score ≥ 8 and the Clinical Global Impressions-Severity Score (CGI-S) ≥ 3 (for bipolar spectrum disorder group); if taking psychotropic medications, or daily multivitamin or vitamin supplement, stably medicated (same dose for 4 weeks prior to enrolment and willing to do it within the study) Exclusion: history of an uncontrolled general medical disorder; history of neurological illness, schizophrenia, or psychosis; history of head trauma with loss of consciousness; substance dependence; suicidal or homicidal ideation; contraindications to magnetic resonance imaging; disorder that would interfere with the action, absorption, distribution, metabolism or excretion of vitamin D3, that might pose a safety concern, or interfere with the accurate assessment of safety and efficacy; axis I diagnosis or a family history of a mood disorder in a first degree (for control group)
[46]	54 (37)	16.0 ± 1.8	Inclusion: depression diagnosed Exclusion: -

ADHD—Attention Deficit Hyperactivity Disorder; BMI—Body Mass Index; DSM—Diagnostic and Statistical Manual of Mental Disorders; *—data provided on request.

**Table 4:** The exposure, intervention and outcome of the included intervention studies.

Ref.	Exposure	Applied Intervention	Outcome	Psychological Measure
[37]	25(OH)cholecalciferol level in blood	Vitamin D supplementation (100 µg vs. 175 µg per day) for 3 months	Health-Related Quality of Life	PROMIS pediatric short forms: depressive symptoms, fatigue, pain, mobility, peer relationships, and upper-extremity function
[41]	Daily vitamin D intake (Vit D-Food record (FR)) 25(OH)cholecalciferol level in blood	Vitamin D supplementation (25 µg per day vs. placebo) for 3 months	Mental health	Strengths and Difficulties Questionnaire—Teacher Version (SDQT), Strengths and Difficulties Questionnaire—Parent Version (SDQP)
[42]	25(OH)cholecalciferol level in blood	Vitamin D supplementation (1250 µg once a week) for 9 weeks in intervention phase	Depressive symptoms Aggressive disorder	Beck Depression Inventory—Persian version (BDI Persian) Buss-Perry Aggression Questionnaire—Persian version (BPAQ)
[43]	25(OH)cholecalciferol level in blood	Vitamin D supplementation (38 µg per day) vs. control for 3 months	Self-perception of mental health	Youth Self-report version of the Child Behavior Checklist (YSR-CBCL)
[44]	25(OH)cholecalciferol level in blood	Ergocalciferol supplementation, depending on the vitamin D status (125 µg per day for patients of 25(OH)D concentration of 50–72.5 nmol/L vs. 1250 µg per week for < 50 nmol/L) for 3 months	Sleep patterns and sleep problems	Children’s Sleep Habits Questionnaire (CSHQ)
[45]	25(OH)cholecalciferol level in blood	Vitamin D supplementation (50 µg per day) for 2 months	Mood Depression Suicidal incident	Young Mania Rating Scale (YMRS) Children’s Depression Rating Scale (CDRS) Columbia-Suicide Severity Rating Scale (CSSR-S)
[46]	25(OH)cholecalciferol level in blood	Vitamin D supplementation depending on the status (for patients of 25(OH)D <60 nmol/L): 100 µg per day for 1 month followed by 50 µg per day for 2 months	Well-being Mood	WHO-5 Well-being scale Mood and Feelings Questionnaire—Short Version (MFQ-S)

**Table 5:** The findings presented in the intervention studies included to the systematic review.

Ref.	Observation	Conclusion
[37]	In subjects with SS sickle cell disease, significant reductions in pain, fatigue, and depressive symptoms and improved upper-extremity function were observed. In healthy subjects, significant reductions in fatigue and improved upper-extremity function were observed.	Daily high-dose vitamin D supplementation for African American children with SS sickle cell disease improved HRQL.
[41]	The mean scores of the SDQP and SDQT showed a significant difference in the two groups after intervention.	Vitamin D supplementation improves some behavioral problems.
[42]	There was a significant reduction on mild, moderate, and severe depression score. However, vitamin D supplementation had no significant effect on aggression score.	Results suggest that supplementation with vitamin D may improve depressive symptoms among adolescent girls, as assessed by questionnaire, but not aggression score.
[43]	Multivariate data analysis showed that participants with low vitamin D status scored worse on the Tower of London tests and the more difficult sub-tasks on the Tower of Hanoi tests. They also had a tendency to report higher frequency of externalizing behavior problems and attention deficit.	The study indicates that vitamin D status in adolescents may be important for both executive functioning and mental health.
[44]	In ASD patients, there was a significant negative correlation between serum 25(OH)D levels and the night waking subscale (r = −0.301, *p* = 0.019). In control patients, there was a significant negative correlation between serum 25(OH)D levels and daytime sleepiness subscales (r = −0.269, *p* = 0.038).	The results indicate that it may be suitable to use 25(OH)D replacement therapy in ASD patients and healthy individuals with sleep disturbances.
[45]	Following an 8 week vitamin D3 supplementation, in BSD patients, there was a significant decrease in YMRS scores (t = −3.66, *p* = 0.002, df = 15) and CDRS scores (t = −2.93, *p* = 0.01, df = 15).	Following an 8 week open label trial with vitamin D3 supplementation, patients with BSD exhibited improvement in their mood in conjunction with their neurochemistry.
[46]	Basal 25(OH)D levels correlated positively with well-being (*p* < 0.05). After vitamin D supplementation, well-being increased (*p* < 0.001) and there was a significant improvement in eight of the nine items in the vitamin D deficiency scale: depressed feeling (*p* < 0.001), irritability (*p* < 0.05), tiredness (*p* < 0.001), mood swings (*p* < 0.01), sleep difficulties (*p* < 0.01), weakness (*p* < 0.01), ability to concentrate (*p* < 0.05) and pain (*p* < 0.05). There was a significant amelioration of depression according to the MFQ-S (*p* < 0.05).	This study showed low levels of vitamin D in depressed adolescents, positive correlation between vitamin D and well-being, and improved symptoms related to depression and vitamin D deficiency after vitamin D supplementation.

ASD—Autism Spectrum Disorder; BSD—Bipolar Spectrum Disorders; CDRS—Children’s Depression Rating Scale; HRQL—Health-Related Quality of Life; MFQ-S—Mood and Feelings Questionnaire—Short Version; SDQP—Strengths and Difficulties Questionnaire—Parent Version; SDQT—Strengths and Difficulties Questionnaire—Teacher Version; YMRS—Young Mania Rating Scale.

**Table 6:** The basic characteristics of the included observational studies, including study design, location, studied group and time.

Ref	Authors, Year	Design of Study	Location	Studied Group	Time
[47]	Robinson et al., 2019	Prospective cohort study within the Bogota School Children Cohort	Colombia/Bogota	Children aged 5–12 years randomly selected from primary public schools in Bogota	February 2006; 2011–2015 (follow-up)
[48]	Bai & Dai, 2018	Case-control study	China	Children with stable asthma compared with controls	January 2013–December 2016
[49]	Han et al., 2018	Cross-sectional study	China/Jiaxing	Pediatric patients on chronic hemodialysis or peritoneal dialysis	May 2013–June 2016
[50]	Huang et al., 2018	Cross-sectional study	China/Zaozhuang	Adolescents with or without depressive disorders aged 8–16 years from outpatient pediatric health care centers	March 2016–May 2018
[51]	Petrov et al., 2018	Cross-sectional study within National Institute of Mental Health (NIMH) Longitudinal Assessment of Manic Symptoms (LAMS) study	United States of America (USA)	Adolescents enrolled in the NIMH LAMS study, which examines mood symptom changes and elevated symptoms of mania biannually	Not specified
[52]	Yazici et al., 2018	Prospective case-control study	Turkey/Elazığ	Children and adolescents aged 7–15 years with obsessive-compulsive disorder compared with controls	February 2015–January 2016
[53]	Esnafoğlu & Yaman, 2017	Case-control study	Turkey/Ordu	Patients with obsessive-compulsive disorder who attended the children and adolescents psychiatry outpatient clinic	December 2014–February 2016 *
[54]	Husmann et al., 2017	Cross-sectional study based on the population-based and nation-wide German Health Interview and Examination Survey for Children and Adolescents (KiGGS) study	Germany	Children aged 3–17.9 participating in the KIGGS study	May 2003–May 2006
[55]	MacDonald et al., 2017	Retrospective medical chart review	Canada/Edmonton	Children (2–18 years) with and without established mental health diagnosis and with an obesity	2011–2014
[56]	Karabel et al., 2016	Retrospective case-control study	Turkey/Diyarbakır	Patients of Adolescent Clinic and Child Psychiatry Clinic	Not specified
[57]	Schäfer et al., 2016	Cross-sectional study based on the population-based and nation-wide German Health Interview and Examination Survey for Children and Adolescents (KiGGS) study	Germany	Adolescents aged 11–17 participating in the KIGGS study	May 2003–May 2006
[58]	Ataie-Jafari et al., 2015	Cross-sectional study within the Childhood and Adolescence Surveillance and PreventIon of Adult Noncommunicable Disease (CASPIAN III) study	Iran	Children and adolescents aged 10–18 years within CASPIAN III study	2009–2010
[59]	Smith et al., 2014	Cross-sectional study	USA/Buffalo	Children aged 7–17 years with cystic fibrosis	Spring of 2007
[60]	Fazeli et al., 2013	Cross-sectional study	USA/Massachusetts	Adolescents aged of 12–18 years with and without major depressive disorder	September 2007–December 2009
[61]	Gracious et al., 2012	Cross-sectional study	USA/Rochester	Adolescents on acute mental health treatment	October 2008–February 2010
[62]	Tolppanen et al., 2012	Longitudinal Study within the Avon Longitudinal Study of Parents and Children (ALSPAC)	United Kingdom/South West England	Children from the ALSPAC	Children born 1991–1992 observed at the age of 7, 9 and 11 (1998–1999; 2000–2001; 2002–2003)
[63]	Tolppanen et al., 2012	Longitudinal Study within the Avon Longitudinal Study of Parents and Children (ALSPAC)	United Kingdom/South West England	Children from the ALSPAC	Children born 1991–1992 observed at the age of 10 and 13 (2001–2002; 2004–2005)

*—data provided on request.

**Table 7:** The characteristics of the subjects of the included observational studies.

Ref.	Number of Participants (Girls)	Age (Mean with SD/Range)	Inclusion Criteria/Exclusion Criteria
[47]	273 (146)	8.6 ± 1.6 (enrolment) 14.7 ± 1.7 (follow-up)	Inclusion: children aged 5–12 years; randomly selected from primary public schools in Bogota within the Bogota School Children Cohort Exclusion: outside the 11–18 years range at follow-up; missing data
[48]	246	8.5 ± 2.4 for asthma group 8.9 ± 2.8 for control group	Inclusion: diagnosed stable asthma; able to cooperate and comply with pulmonary function tests Exclusion: restrictive ventilation dysfunction; history of calcium supplementation; recently taken vitamin D; serious diseases
[49]	156 (65)	13.8 ± 2.4	Inclusion: Chinese ethnicity; aged 8–18 years; receipt of dialysis therapy for at least 3 months; hospitalization within 14 days (exclusive of a hospital stay for dialysis) Exclusion: severe visual or auditory impairment or mental retardation; renal transplant recipients; history of psychiatric disorders including anxiety and depression; malignancy/leukaemia; chronic hepatic disease; autoimmune diseases or active infections; significant life event unrelated to their renal disease in the past 30 days (e.g., severe illness of a family member, family structure changes, losing a family member, changing the living place); osteoporosis or receiving supplementation with ergocalciferol or cholecalciferol
[50]	270 (145)	12.1 ± 2.6 for male depressive disorder group 11.6 ± 2.8 for male control group 12.0 ± 2.7 for female depressive disorder group 12.3 ± 2.8 for female control group	Inclusion: aged 8–16 years; BMI ranging from the 5th to 95th percentiles; depressive disorders diagnosed by local psychiatrists (for depressive disorder group) Exclusion: suicidal ideation; receiving medical treatments that could affect thyroid function in the preceding 2 months
[51]	36	14.0 ± 2.4 for non-mood disorders group 14.1 ± 1.2 for major mood disorders group 13.9 ± 2.0 for bipolar disorder group	Inclusion: adolescents enrolled in the National Institute of Mental Health (NIMH) Longitudinal Assessment of Manic Symptoms (LAMS) study; mood symptom changes and elevated symptoms of mania biannually Exclusion: -
[52]	119 (45)	11.2 ± 2.8 for obsessive compulsive disorder group 10.9 ± 2.7 for control group	Inclusion: for control group age and sex distribution comparable with obsessive compulsive disorder group Exclusion: The Wechsler Intelligence Scale for Children-Revised (WISC-R) score < 80; comorbid psychiatric disorder according to K-SADS-PL; history of psychotropic drug or substance using; calcium or vitamin D supplementation within the previous 6 months; history of serious head trauma; systemic disease (epilepsy, history of antiepileptic use, clinically active infection, obesity, etc.); abnormal neurological symptoms; pathology in routine biochemical tests; history of corticosteroid use; insufficient collection of blood volume
[53]	82 (42)	14.7 ± 2.3 for obsessive compulsive disorder group 14.2 ± 2.6 for control group	Inclusion: patient of the outpatient clinic for children and adolescent psychiatry at Ordu University Research and Training Hospital; diagnosis according to DSM-V criteria (for obsessive compulsive disorder group); minor issues (for control group) Exclusion: infections; psychotic disorders; diagnosis of mental retardation and developmental disorders; nutritional support product used in the previous year; vegetarians
[54]	9068 (4445)	3.0-17.9	Inclusion: aged 3–17.9 years Exclusion: missing data
[55]	217 (105)	12.0 ± 2.9	Inclusion: patients attending a Pediatric Center for Weight Management Treatment Centre in Alberta, Canada; aged 2–18 years; overweight (BMI between the 85th and 97th percentile) or obesity (BMI above the 97th percentile) Exclusion: missing data *
[56]	138 (69)	12.8 ± 1.9 for depression group 12.7 ± 1.8 for control group	Inclusion: patients of Adolescent Clinic and Child Psychiatry Clinic of the Medical School of Dicle University Exclusion: missing data
[57]	5066 (2481)	14.7 ± 2.0 for males 14.6 ± 2.0 for females	Inclusion: aged 11–17 years Exclusion: missing data
[58]	1095 (527)	14.7 ± 2.6	Inclusion: students; aged 10–18 years; selected randomly by multistage random cluster sampling Exclusion: -
[59]	38 (20)	12.1 ± 3.1	Inclusion: aged 7–17 years; proven diagnosis of cystic fibrosis via sweat test (sweat Cl > 60 mmol/L) Exclusion: no exclusion criteria *
[60]	65 (33)	16.8 ± 1.5 for male major depressive disorder group 16.4 ± 2.0 for male control group 16.6 ± 1.4 for female major depressive disorder group 16.3 ± 2.2 for female control group	Inclusion: outpatients from the Massachusetts General Hospital for Children, BMI between the 5th and 95th percentiles for age; being referred by local psychiatrists (for major depressive disorder group) Exclusion: medical conditions or medications that could affect bone metabolism (including estrogen/progesterone or glucocorticoids) within the preceding 3 months; any other axis I disorder except for co-morbid anxiety; suicidal ideation
[61]	104 (75)	15.4 ± 1.6	Inclusion: patients of the Strong Behavioral Health Child and Adolescent Acute Inpatient Service or Partial Hospitalization Service (CAPHS), Department of Psychiatry, University of Rochester; acute mental health treatment over a 16-month period Exclusion: -
[62]	2413 (1306) *	9.9 ± 1.1	Inclusion: participant of the Avon Longitudinal Study of Parents and Children (ALSPAC) cohort from South West England (single and twin births born between 1 April 1991, and 31 December 1992) Exclusion: any previous behavioral problems at the age of 7 or 9; missing data
[63]	2759	9.8 for vitamin D assessment 10.6 and 13.8 for depressive symptoms assessment	Inclusion: participant of the ALSPAC cohort from South West England (single and twin births born between 1 April 1991, and 31 December 1992) Exclusion: -

BMI—Body Mass Index; DSM—Diagnostic and Statistical Manual of Mental Disorders; K-SADS-PL—Kiddie Schedule for Affective Disorders and Schizophrenia for School-Age Children-Present and Lifetime Episode; *—data provided on request.

**Table 8:** The exposure, intervention and outcome of the included observational studies.

Ref.	Exposure	Outcome	Psychological Measure
[47]	25(OH)cholecalciferol level in blood	Behavior problems	Child Behavior Checklist (CBCL) Youth Self-Report (YSR)
[48]	25(OH)cholecalciferol level in blood	Quality of life	Activity of Daily Living (ADL) score Medical Research Council (MRC) score
[49]	25(OH)cholecalciferol level in blood	Anxiety symptoms	Screen for Child Anxiety Related Emotional Disorders—Chinese version (SCARED)
[50]	25(OH)cholecalciferol level in blood	Depressive symptoms	1-item self-reported mental health questionnaire (feeling despair and/or sad continuously for more than 2 weeks to a point that interferes with normal study and life)
[51]	25(OH)cholecalciferol level in blood	Bipolar disorder and major depressive disorder	Kiddie Schedule for Affective Disorders and Schizophrenia for School-Age Children-Present and Lifetime Episode —Version Plus (K-SADS-PL-W)
[52]	25(OH)cholecalciferol level in blood	Psychopathologies Severity of obsessive compulsive disorder Depression	Kiddie Schedule for Affective Disorders and Schizophrenia for School-Age Children-Present and Lifetime Version (K-SADS-PL) for DSM-IV diagnostic criteria Children’s Yale Brown Obsession Compulsion Scale (CYBOCS) Children’s Depression Inventory (CDI)
[53]	25(OH)cholecalciferol level in blood	Severity of obsessive compulsive disorder Depression Anxiety	Yale-Brown Obsessive Compulsive Scale (Y-BOCS) Children’s Depression Inventory (CDI) State-Trait Anxiety Inventory 1 and 2 (STAI-1 and STAI-2)
[54]	25(OH)cholecalciferol level in blood	Mental health	Strengths and Difficulties Questionnaire (SDQ)
[55]	25(OH)cholecalciferol level in blood	Mental health	DSM-V criteria
[56]	25(OH)cholecalciferol level in blood	Depression	Depression Scale for Children (DSC)
[57]	25(OH)cholecalciferol level in blood	Health-Related Quality of Life (HRQoL) Level of distress	Children’s Quality of Life Questionnaire (Kinder-Lebensqualitatsfragebogen, KINDL-R) Strengths and Difficulties Questionnaire (SDQ)
[58]	25(OH)cholecalciferol level in blood	Psychiatric distress and violence behaviors	Global School-based Student Health Survey (GSHS) Questions about violence
[59]	25(OH)cholecalciferol level in blood	Depression	Children’s Depression Inventory (CDI)
[60]	25(OH)cholecalciferol level in blood	Depression	Children’s Depression Inventory (CDI) Children’s Depression Rating Scale—Revised (CDRS-R)
[61]	25(OH)cholecalciferol level in blood	Severity of illness, defined by presence of psychotic features	Clinical DSM-IV diagnose
[62]	25(OH)cholecalciferol level in blood	Mental health	Strengths and Difficulties Questionnaire (SDQ)
[63]	25(OH)cholecalciferol level in blood	Depressive symptoms	Mood and Feelings Questionnaire (MFQ)

DSM—Diagnostic and Statistical Manual of Mental Disorders.

**Table 9:** The findings presented in the observational studies included to the systematic review.

Ref.	Observation	Conclusions
[47]	Vitamin D deficiency was associated with an adjusted 6.0 (95% CI: 3.0, 9.0) and 3.4 (95% CI: 0.1, 6.6) units higher Child Behavior Checklist and Youth Self-Report externalizing problems scores, respectively, and an adjusted 3.6 (95% CI: 0.3, 6.9) units higher Child Behavior Checklist internalizing problems scores. The prevalence of clinical total externalizing problems was 1.8 (95% CI: 1.1, 3.1) times higher in children with vitamin D deficiency than that in children without vitamin D deficiency.	Vitamin D deficiency in middle childhood is related to behavior problems in adolescence.
[48]	Serum 25(OH)D levels were positively correlated with ADL score in children with stable asthma, and negatively correlated with MRC score.	Increased serum 25(OH)D levels reflect good QoL in children with stable asthma.
[49]	Serum levels of 25(OH)D were significantly lower in patients with anxiety than in normal controls (19.4 ± 10.3 vs. 38.6 ± 15.5 ng/mL, *p* < 0.001). Serum 25(OH)D levels (≤15.0 ng/mL) were independently associated with the existent of anxiety in children and adolescents receiving dialysis (OR 4.650, 95% CI: 1.663–13.001, *p* = 0.003).	Low serum levels of vitamin D are independently associated with anxiety among children and adolescents on dialysis, which needs to be confirmed in future experimental and clinical studies.
[50]	Patients with depressive disorder had lower concentrations of 25(OH)D (*p* < 0.005) than control participants, in both male and female cohorts. However, serum 25(OH)D concentration did not significantly correlate with depressive symptoms.	Adolescents with depressive disorder have markedly lower serum 25(OH)D concentrations than control patients. This relationship is positively associated with disease progression, suggesting possible nutritional intervention measures for neuroprotection.
[51]	There was no difference between serum vitamin D concentrations in participants from non-mood control, major mood disorders, and bipolar disorder groups.	There was no difference between serum vitamin D concentrations in participants from non-mood control, major mood disorders, and bipolar disorder groups.
[52]	Vitamin D levels were lower in patients diagnosed with OCD (15.88 ± 6.96 ng/mL) when compared to healthy controls (18.21 ± 13.24 ng/mL), but the difference was not statistically significant (*p* = 0.234). A negative correlation was found between serum 25(OH)D levels and obsession scale scores in CYBOCS.	The vitamin D levels of newly diagnosed OCD cases were lower than that of healthy controls; however, the difference was not statistically significant. The study does not support presence of a significant association between serum vitamin D levels and OCD.
[53]	Significantly lower levels of vitamin D in the patient group compared to control group (*p* < 0.001) were observed.	Vitamin D deficiency can play a role in the etiology of OCD.
[54]	There were inverse associations between 25(OH)D concentrations and the subscales emotional problems, peer relationship problems and the total difficulties score in both genders after adjustment for potential confounders. The strongest associations were observed in the older subsample for parent-ratings in boys and self-ratings in girls. In the younger subsample the associations were less strong and no longer evident after adjustment for potential confounders such as migration background, socioeconomic status and frequency of playing outside.	Based on the large-scale cross-sectional study in a German population-based sample of children and adolescents inverse associations between 25(OH)D concentrations and both parent- and self-rated SDQ scores of the total difficulties scale and different subscales with the strongest association in the subsample aged 12–18 years for both genders were detected.
[55]	No relationships between mental health parameters (type or total) and vitamin D status were observed.	The influence of vitamin D status on mental health may extend beyond mental health disease type to disease severity, because disease expression may change with overall child development.
[56]	Negative correlation was found between the vitamin D levels and depression score in the group with depression (r = −0.368; *p* = 0.03).	Even if clinical depression is absent, the frequency of depressive symptoms is increased with decreased vitamin D levels, independent of other factors. Maintaining vitamin D support during adolescence, as with the first year of life, is necessary for both the prevention and treatment of depression.
[57]	Bivariate analyses demonstrated a significant positive association between 25(OH)D and HRQoL for both self- [estimate (E) = 0.82, 95% CI 0.35–1.30, *p* = 0.001] and parent ratings (E = 1.33, 95% CI 0.83–1.83, *p* < 0.001). In addition, negative correlations between 25(OH)D and self (E = −0.34, 95 % CI -0.58 to −0.11, *p* = 0.005) and parent-reported total SDQ scores (E = −0.70, 95% CI −1.03 to −0.37, *p* < 0.001) were found. Generalized linear models adjusted for age, sex, BMI, systolic blood pressure, migration background, socio-economic status, and sedentary screen time confirmed that 25(OH)D independently and significantly predicted better HRQoL (*p* ≤ 0.004).	These findings linking 25(OH)D to better well-being in a nationally representative sample of German children and adolescents suggest beneficial effects of vitamin D on mental health. However, recommendations for vitamin supplementation in healthy children and adolescents are not warranted from the data.
[58]	The prevalence of self-reported anger, anxiety, poor quality sleep, sadness/depression, and worry was significantly lower in vitamin D sufficient participants compared with their other counterparts. The odds of reporting anger, anxiety, poor quality sleep, and worry, increased approximately 1.5 to 1.8 times in vitamin D insufficient compared with normal children and adolescents. Risk estimates indicated that vitamin D insufficient and deficient subjects had higher odds of reporting worry compared to normal vitamin D group [OR = 2.417 (95% CI: 1.483–3.940) for vitamin D insufficient students, and OR = 2.209 (95% CI: 1.351–3.611) for vitamin D deficient students] (*p*-trend = 0.001). Violence behaviors did not show any association with vitamin D status (*p* > 0.05).	Some psychiatric distress such as anger, anxiety, poor quality sleep, depression, and worry are associated with hypovitaminosis D in adolescents.
[59]	Serum 25(OH)D was negatively associated with CDI scores (r = −0.55, *p* < 0.001), and the group of patients with insufficient 25(OH)D levels indeed reported significantly more depressive symptoms (t = 4.26; *p* < 0.001).	The 25(OH)D insufficiency was associated with depressive symptoms in this cohort of youth with CF. Future rigorous studies investigating vitamin D and depression in CF are warranted with larger sample sizes using confirmatory methods to diagnose depressive disorders.
[60]	The 25(OH)D did not differ in girls with MDD compared to controls, even after adjusting for BMI, lean mass and bone age. Vitamin D levels were not significantly different in MDD compared to controls even after adjusting for BMI. Vitamin D was significantly higher in girls with MDD as compared to controls (MDD: 33.5 ± 8.1 versus healthy controls: 22.5 ± 8.0 ng/mL; *p* < 0.001), and this difference remained statistically significant after adjusting for BMI (*p* = 0.001).	Vitamin D was significantly higher in girls with MDD as compared to controls, but did not differ for other comparisons.
[61]	Adolescents with psychotic features had lower vitamin D levels than those without (20.4 ng/mL vs. 24.7 ng/mL; *p* = 0.04, 1 df). The association for vitamin D deficiency and psychotic features was substantial (OR 3.5; 95% CI 1.4–8.9; *p* < 0.009).	Vitamin D deficiency and insufficiency are both highly prevalent in adolescents with severe mental illness.
[62]	Higher 25(OH)D3 concentrations were weakly associated with lower risk of prosocial problems (fully adjusted OR 95% CI 0.85 (0.74, 0.98)). Serum 25(OH)D3 or 25(OH)D2 concentrations were not associated with other subscales of SDQ or total difficulties score after adjusting for confounders and other measured analytes related to vitamin D.	The findings do not support the hypothesis that 25(OH)cholecalciferol status in childhood has important influences on behavioral traits in humans.
[63]	Higher concentrations of 25(OH)D3 assessed at mean age 9.8 years were associated with lower levels of depressive symptoms at age 13.8 years [adjusted RR; 95% CI: 0.90 (0.86–0.95)], but not at age 10.6 years [adjusted RR 95% CI: 0.98 (0.93–1.03)] and with increased odds of decreasing symptoms between age 10.6 and 13.8 years [adjusted RR 95% CI: 1.08 (1.01–1.16)]. Serum 25(OH)D2 concentrations were not associated with depressive symptoms.	The association between vitamin D level and depressive symptoms is independent of a wide range of potential confounding factors, and appears to be stronger with greater time separation between assessment of 25(OH)D and assessment of depressive symptoms.

ADL—Activity of Daily Living; BMI—Body Mass Index; CDI—Children’s Depression Inventory; CF—Cystic Fibrosis; CI—Confidence Interval; CYBOCS—Children’s Yale Brown Obsession Compulsion Scale; HRQoL—Health-Related Quality of Life; MDD—Major Depressive Disorder; MRC—Medical Research Council; OCD—Obsessive-Compulsive Disorder; OR—Odds Ratio; QoL—Quality of Life; RR—Risk Ratio; SDQ—Strengths and Difficulties Questionnaire.

**Table 10:** The summary of observations and conclusions for the included studies of association between vitamin D and mental health, with the total Newcastle-Ottawa Scale (NOS) score.

Ref.	Potential Influence of Vitamin D	Results Supporting/Inconclusive/Not Supporting Positive Association between Vitamin D Intake and Mental Health *	Quality **
[37]	Reduced pain, fatigue, and depression, as well as improved upper-extremity function	Supporting	3
[41]	Reduced some behavioral problems	Supporting	5
[42]	Reduced depression	Supporting	4
[43]	Reduced externalizing behavior problems and attention deficit and improved cognition	Supporting	3
[44]	Reduced sleep disturbances	Supporting	4
[45]	Reduced depression and mania	Supporting	4
[46]	Reduced depression, irritability, tiredness, mood swings, sleep difficulties, weakness and pain, as well as improved well-being and ability to concentrate	Supporting	3
[47]	Reduced externalizing and internalizing problems	Supporting	7
[48]	Improved quality of life	Supporting	3
[49]	Reduced anxiety	Supporting	5
[50]	Reduced/not reduced depression (depending on analysis)	Inconclusive	4
[51]	No effect on bipolar disorder and major depressive disorder	Not supporting	2
[52]	No effect on obsessive compulsive disorder, but reduced obsession component	Inconclusive	4
[53]	Reduced obsessive compulsive disorder	Supporting	5
[54]	Reduced emotional problems and peer relationship problems	Supporting	5
[55]	No effect on mental health	Not supporting	5
[56]	Reduced depression	Supporting	3
[57]	Reduced distress and improved quality of life	Supporting	5
[58]	Reduced anger, anxiety, depression and worry, as well as improved quality of sleep	Supporting	5
[59]	Reduced depression	Supporting	4
[60]	Reduced/not reduced depression (depending on analysis)	Inconclusive	5
[61]	Reduced psychotic features	Supporting	5
[62]	Reduced prosocial problems	Supporting	8
[63]	In longer term reduced depression	Supporting	7

* Supporting—vitamin D associated with lower risk of mental health problems; not supporting—vitamin D not associated with lower risk of mental health problems; inconclusive—no clear association between vitamin D and risk of mental health problems; ** total score for the NOS.
